# Outbreak of *Pseudomonas aeruginosa* producing VIM carbapenemase in an intensive care unit and its termination by implementation of waterless patient care

**DOI:** 10.1186/s13054-021-03726-y

**Published:** 2021-08-19

**Authors:** Gaud Catho, R. Martischang, F. Boroli, M. N. Chraïti, Y. Martin, Z. Koyluk Tomsuk, G. Renzi, J. Schrenzel, J. Pugin, P. Nordmann, D. S. Blanc, S. Harbarth

**Affiliations:** 1 Infection Control Program, WHO Collaborating Center for Patient Safety, Faculty of Medicine, Geneva University Hospitals, Rue Gabrielle Perret-Gentil, 4, CH-1205 Geneva, Switzerland; 2 Division of Critical Care, Faculty of Medicine, Geneva University Hospitals, Geneva, Switzerland; 3 Bacteriology Laboratory, Faculty of Medicine, Geneva University Hospitals, Geneva, Switzerland; 4 Emerging Antibiotic Resistance Unit, Medical and Molecular Microbiology, Department of Medicine, Faculty of Science and Medicine, University of Fribourg, Fribourg, Switzerland; 5 Swiss National Reference Center for Emerging Antibiotic Resistance, Fribourg, Switzerland; 6 Service of Hospital Preventive Medicine, Lausanne University Hospital, University of Lausanne, Lausanne, Switzerland

**Keywords:** *Pseudomonas aeruginosa*, VIM, Carbapememase, Sink, Waterless, Outbreak, Aquatic reservoir, cgMLST

## Abstract

**Background:**

Long-term outbreaks of multidrug-resistant Gram-negative bacilli related to hospital-building water systems have been described. However, successful mitigation strategies have rarely been reported. In particular, environmental disinfection or replacement of contaminated equipment usually failed to eradicate environmental sources of *Pseudomonas aeruginosa.*

**Methods:**

We report the investigation and termination of an outbreak of *P. aeruginosa* producing VIM carbapenemase (PA-VIM) in the adult intensive care unit (ICU) of a Swiss tertiary care hospital with active case finding, environmental sampling and whole genome sequencing (WGS) of patient and environmental strains. We also describe the implemented control strategies and their effectiveness on eradication of the environmental reservoir.

**Results:**

Between April 2018 and September 2020, 21 patients became either infected or colonized with a PA-VIM strain. For 16 of them, an acquisition in the ICU was suspected. Among 131 environmental samples collected in the ICU, 13 grew PA-VIM in sink traps and drains. WGS confirmed the epidemiological link between clinical and environmental strains and the monoclonal pattern of the outbreak. After removing sinks from patient rooms and implementation of waterless patient care, no new acquisition was detected in the ICU within 8 months after the intervention.

**Discussion:**

Implementation of waterless patient care with removal of the sinks in patient rooms was successful for termination of a PA-VIM ICU outbreak linked to multiple environmental water sources. WGS provides highly discriminatory accuracy to investigate environment-related outbreaks.

**Supplementary Information:**

The online version contains supplementary material available at 10.1186/s13054-021-03726-y.

## Background

*Pseudomonas aeruginosa* is an important cause of hospital-acquired infections, affecting predominantly fragile or immunocompromised patients [1–3]. A major factor in its prominence as a pathogen is its intrinsic resistance to antibiotics and disinfectants [4]. Increasing incidence of multidrug resistant *P. aeruginosa*, in particular carbapenemase-producing strains, is a global concern [5]. The Verona Integron-encoded Metallo-beta-lactamase (VIM), the most widespread Metallo-beta-lactamase (MBL) produced by *P. aeruginosa*, hydrolyses all classes of beta-lactams except monobactams and cefiderocol [6], making therapeutic options in case of infection very limited [7]. Carbapenem-resistant *P. aeruginosa* has therefore been ranked as critical-priority bacteria in the WHO priority list of antibiotic resistant bacteria [8]. In this context, prevention of acquisition and spread of these strains is a priority.

Hospital water and water-related devices may serve as a reservoir of healthcare-associated pathogens [9]. Several clinically important Gram-negative bacterial species, including *P. aeruginosa* are well adapted to colonize the biofilms of water systems, and their presence has been associated with sporadic infections and outbreaks in hospitalized patients [10]. Eradication of water reservoirs is particularly challenging and mitigation strategies have been rarely reported to be successful in the long-term [11]. We report the investigation of an outbreak of VIM-53 positive *P. aeruginosa* (PA-VIM) in the intensive care unit (ICU) of a Swiss tertiary care hospital with the first case being detected in March 2018, the identification of an environmental aquatic source with molecular characterisation and genomic analysis confirming the monoclonal pattern of the outbreak and the effect of the mitigation strategy. In accordance with the ORION recommendations for outbreak reporting [12], we provide a detailed description of the outbreak and its source, the containment measures that were implemented, and the follow-up of colonized or infected patients.

## Methods

### Setting

**Fig. 1:**
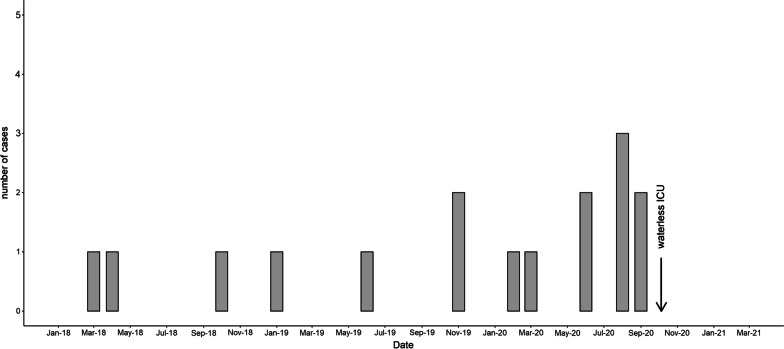
Epidemic curve of cases with infection and/or acquisition of *Pseudomonas aeruginosa* VIM in the intensive care unit (ICU). Between March 2018 and September 2020, 16 patients were newly colonized and/or infected with a *P. aeruginosa* producing VIM strain during their ICU stay. In October 2020, waterless patient care was implemented in the ICU with no new acquisition event in the ICU during the next 8 months

Geneva University Hospitals (HUG) is a large tertiary care hospital located in Geneva, Switzerland, with approximately 1900 patient beds. The adult ICU is a mixed medical-surgical ICU, comprised of 2 subunits with a total of 30 beds and providing care for approximately 2500 patients annually with an average length of stay of 4 days. One ward contains only single patient rooms with a sink and sewage disposal in each patient room (Fig. 1). The other ward contains patient rooms with 3–4 beds and for each patient room, a medication preparatory area with a sink, and a separated soiled utility room with a sink and a sewage disposal. The incidence of carbapenemase-producing *Enterobacteriacae* or non-fermentative Gram-negative bacteria detected in our institution in 2018, when the outbreak started, was low with 1.3 cases per 1000 patient days. From March 9 to May 19, 2020, the ICU expanded its capacity to 110 beds by beds densification and surface extension to admit the 1st wave of severe COVID-19 patients; the single patient rooms were reserved to COVID negative and/or patients infected or colonised by multidrug resistance organisms (MDRO) [13].

### Infection control measures

Preventive measures were based on recent evidence and guidelines [14]. Weekly screening for intestinal carriage of MDRO was performed among all ICU patients. Targeted screening was performed at admission for patients presenting specific risk profiles [15]. In September 2020, due to the detection of further PA-VIM acquisition events, the ICU screening strategy was intensified with screening for MDRO upon admission, discharge and twice weekly for all patients present on the day of screening. In addition to rectal swabs, sampling of clinical sites (tracheal aspirations, urines, wounds) was encouraged. The enhanced screening was maintained for three months after the last case was detected. Detection of carbapenemase-producing (CP) *P. aeruginosa* prompted enhanced infection control procedures including contact precautions, single bed bedroom isolation and environmental chlorine cleaning. Patient status was marked with an alert in the electronic health record. Contact precautions and specific environmental cleaning procedures were maintained until 5 negative samples. As part of a ventilator-associated pneumonia (VAP) prevention bundle, patients likely to be ventilated more than 48 h receive three times per day selective oral-pharyngeal decontamination (SOD) with colistin, tobramycin and nystatin [16]. Daily SOD administration was maintained as long as the patient remained under mechanical ventilation. In case of newly identified colonisation by a MDR bacteria, the SOD regimen was interrupted. Due to a concomitant endemic problem with *Serratia marcescens* in the ICU detected in 2017 with a suspected water reservoir, several preventive interventions were already implemented in 2018 to mitigate contamination of sinks and reduce transmission of Gram-negative bacteria from potentially colonized sinks [17]. Educational rounds to reinforce compliance with hand hygiene, proper use of gloves and aseptic care procedures while using water were regularly performed. Mitigation strategies focused on re-enforced training of nursing staff on hand hygiene. Modification of behaviors to minimize drain colonisation were implemented, including limitation of the use of sinks for hand hygiene when specifically indicated only, procedures for patient bathing, separation of non-contaminated and contaminated areas and tasks, dedicated storage space > 1 m from sinks, and no use of sinks dedicated to direct care to the patient for hand washing. No disinfection of the sinks was performed.

### Case definition

A case was defined as a patient with a clinical or screening sample positive for PA-VIM. A case was defined as definitely nosocomial if the patient was negative on a previous sample from the same body site during the same hospital stay. A case was defined as probably nosocomial when the patient had no previous negative sample during the same hospital stay and the first positive sample was retrieved more than 48 h after patient admission. A case was attributed to the ICU when the first positive sample was collected more than 48 h after the patient admission in the ICU, either during or after the stay in the ICU. All positive cases were followed until discharge or death.

### Environmental sampling

Environmental sampling of water sources in the ICU was conducted between September and October 2020. Altogether, 131 environmental samples were collected. The samples were collected on sink drains (72), sink traps (36), sink elbows (3), washer decontaminators (8), water (6), water collected through bacterial filter (4), wipes (1) and ultrasound gel (1).

### Bacterial identification, molecular characterization and genomic analysis

Clinical specimens, rectal swabs or stool samples and environmental specimens were inoculated on selective media (ESBL ChromID, McConkey and ChromID OXA-48). Specific search for *P. aeruginosa* was systematically performed. Bacterial isolates were identified by matrix-assisted laser desorption ionization time-of-flight mass spectrometry (MALDI-TOF MS, MALDI-Biotyper Bruker Daltonics). Antimicrobial susceptibility testing was performed in accordance with European Committee on Antimicrobial Susceptibility Testing guidelines by disk susceptibility testing. MICs for multidrug resistant isolates were determined by liquid susceptibility testing (Sensititre, ThemoFisher®). Selected isolates identified as carbapenem resistant by disk testing were additionally screened locally for carbapenemase production via polymerase chain reaction (PCR) (Amplex, eazyplex® SuperBug CRE for the detection of NDM, OXA-48, OXA-181 and VIM carbapenemase genes and home-made PCR for the detection of SME, IMP, GES, SPM, SIM, GIM). Strains were sequenced using the Illumina MiSeq platform. Sequence reads were analyzed using BioNumerics™ (version 7.6.3, created by bioMérieux, available at http://www.applied-maths.com). Core genome Multi Locus Sequence Typing (cgMLST) was performed as previously described [18].

### Ethical considerations

This work was classified as service evaluation and outbreak investigation, and was therefore exempted from Ethics Committee Review.

## Results

### Outbreak description

Between March 2018 and September 2020, 21 patients newly colonised or infected with PA-VIM were identified at HUG. All PA-VIM strains were resistant to colistin. All cases were considered as definitely nosocomial or probably nosocomial, except one that was detected positive at admission screening but with several prior hospital stays; thus, prior hospital acquisition cannot be ruled out. Five patients never stayed in the ICU; however, a possible cross-transmission event outside the ICU with a positive case from the ICU was suspected for one of them; for the four other patients the source of acquisition could not be established. Acquisition was considered linked to the ICU for 16 patients out of 21 (76.2%) (Fig. 1). For 12 patients the first positive sample was collected during ICU stay. For 4 other patients, the detection of the first positive sample occurred after an ICU stay. The attack rate in the ICU for the entire period (from March 2018 to December 2020) was 2.3 /1000 admissions, with the highest rate occurring in August 2020 (18.4/1000 admissions). A cross-transmission event was suspected for one patient (case 11 in Table 1) because of the temporal and spatial link with another case (case 10) in the ICU.

**Table 1:** Demographics and clinical characteristics of cases with an ICU-linked acquisition with date and site of isolation of VIM-PA (*N* = 16)

Patient	Gender	Age (years)	Date of isolation of VIM-PA	Culture site	Initial site of infection or colonisation	Final outcome	Selective oral decontamination	Immunosupression/comorbidities	Site of acquisition
1	M	61	2018-03-16	Urine	Urine colonisation	Discharge	no	Chronic kidney disease	Possible ICU
2	F	55	2018-04-09	Urine	Urine colonisation	Discharge	no	Renal transplant	Possible ICU
3	M	67	2018-10-15	Perianal swab	Intestinal colonisation	Discharge	no	Severe aplastic anemia	ICU
4	F	67	2019-01-21	Wound	Wound colonisation	Discharge	no	Asthma, rheumatoid polyarthritis	Possible ICU
5	M	33	2019-06-16	Tracheal secretion, perianal swab	Respiratory tract colonisation and intestinal colonisation	Discharge	no	Tetraparesis	ICU
6	M	61	2019-11-11	Urine, wound, perianal swab	Urine and intestinal colonisation and sacral pressure ulcer	Discharge	no	HIV, Lymphoma B	ICU
7	M	66	2019-12-31	Urine	Urine colonisation	Discharge	no	Renal transplant	Possible ICU
8	M	67	2020-02-03	LBA, perianal swab	Peritonitis, intestinal colonisation	Discharge	yes		ICU
9	F	35	2020-03-21	Blood	Bloodstream infection, septic choc	Death	yes	Renal transplant, hematologic malignancy	ICU
10	M	56	2020-06-07	Perianal swab, urine, sputum	Intestinal and urine colonisation	Discharge	no	Chronic cardiac disease	ICU
11	M	75	2020-06-28	Perianal swab	Intestinal colonisation	Death	no		ICU
12	M	68	2020-08-01	Urine, blood	Urinary tract infection, bloodstream infection	Discharge	yes	Renal transplant	ICU
13	F	16	2020-08-09	Urine	Intestinal and urine colonisation	Discharge	yes	Renal and liver transplant	ICU
14	F	43	2020-08-26	Wound sacral	Intestinal colonisation and wound (sacral pressure ulcer)	Discharge	no	Denutrition	ICU
15	F	75	2020-09-07	Perianal swab	Intestinal colonisation	Discharge	yes		ICU
16	M	53	2020-09-14	Perianal swab, urine	Intestinal and respiratory tract colonisation, orchi-epididymitis	Discharge	no	Stem cell transplant	ICU

VIM-PA: *Pseudomonas aeruginosa* producing VIM carbapenemase

Demographics and clinical characteristics of the 16 patients with suspected or confirmed ICU acquisition are summarized in Table 1. Median age was 61 years (IQR, 50.5–67); 10 (62.5%) were male. Five of them (31.3%) were infected and 11 (68.7%) were colonized only. Sites of colonisation were intestinal colonisation for 10 (66.7%), urinary colonisation for 6 (37.5%), respiratory tract colonisation for 2 (12.5%) and wound colonisation for 3 (18.7%). Six patients had more than one site colonised. One patient presented a bloodstream infection and one died during the course of his infection. Another case died in the context of undocumented sepsis, with unclear attributability to PA-VIM. Five patients received selective oral decontamination (SOD) for VAP prevention.The median time lag between start of the SOD and first documentation of PA-VIM was 16 days (IQR 9–18). No decolonisation regimen was offered to patients, due to the lack of evidence of decolonisation strategies for carbapenemase producing bacteria [19, 20].

### Environmental investigations

**Fig. 2:**
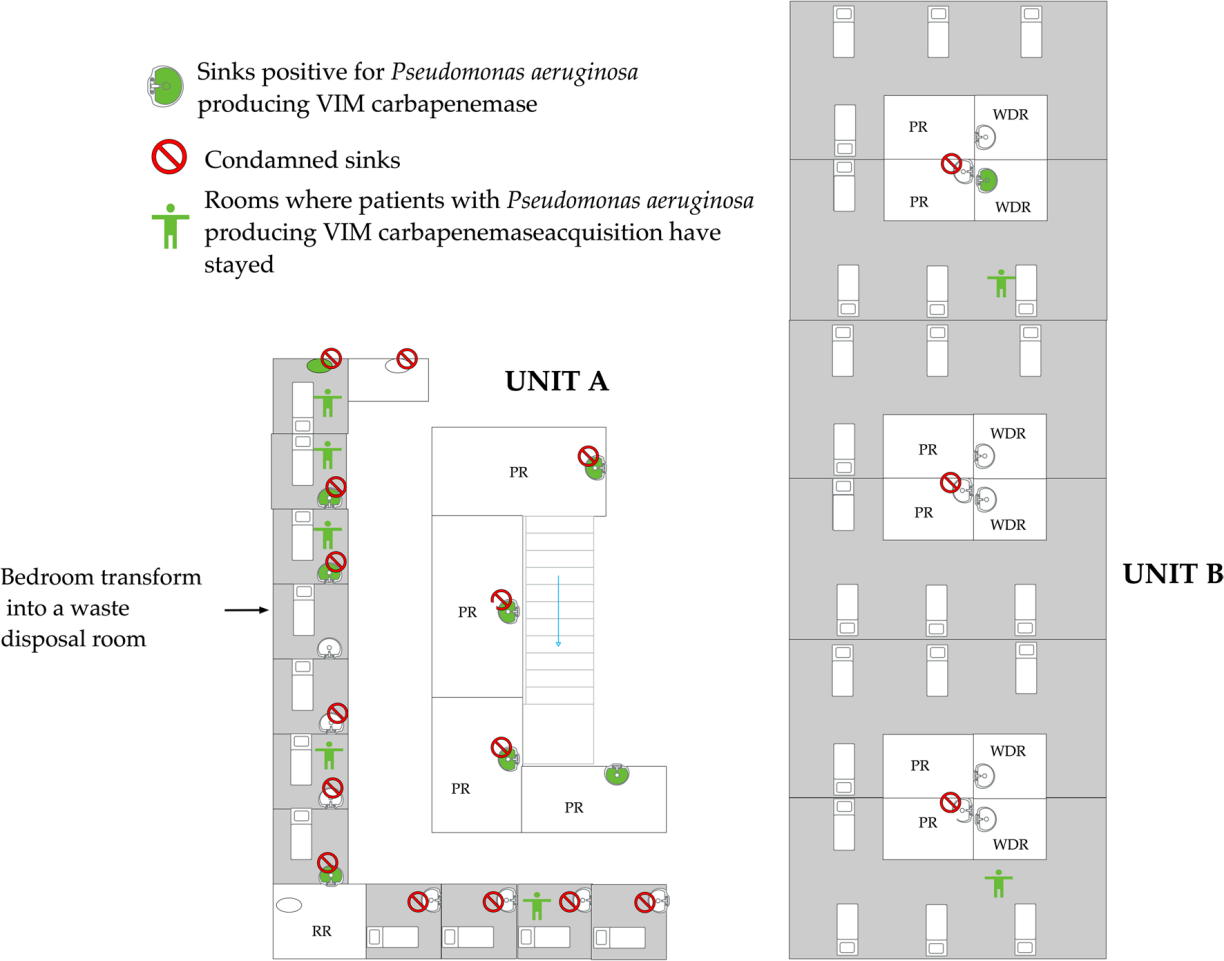
Map of the intensive care unit with the two subunits A and B. Sinks with samples positive for *Pseudomonas aeruginosa* VIM are filled in green. Sinks condemned are marked with a red forbidden sign. Bedrooms who hosted patients with newly *P. aeruginosa* VIM acquisition are marked with a green human symbol. PR: preparation room, WDR: waste disposal room, RR: rest room for healthcare workers

Among the 131 environmental samples collected, 16 were positive for PA-VIM (12.2%). Among the positive samples, 11 samples were taken from sink traps, 4 from sink strainers and 1 from a washer disinfector. Location of the positive and negative samples in the ICU are shown in Fig. 2.

### Sequencing results

**Fig. 3:**
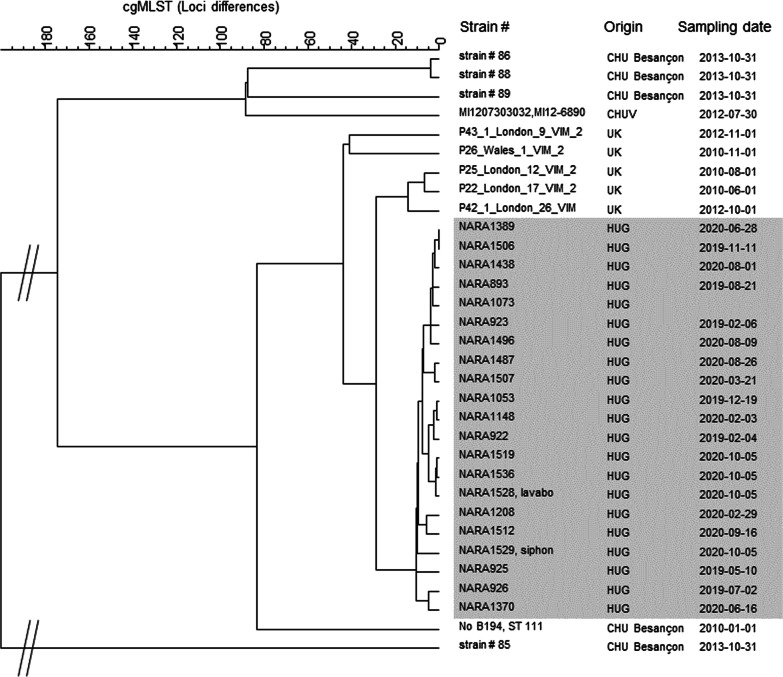
UPGMA similarity tree based on cgMLST analysis of 32 ST111 strains of *Pseudomonas aeruginosa*. The tree includes 19 clinical strains and 2 environmental strains (sinks drains) from HUG, 5 strains from UK, 5 strains from Besançon (France) and 1 strain from Lausanne (Switzerland). The strain number, its origin and date of sampling are mentioned. The 21 strains from HUG belong to the same cluster*.* From the 19 clinical strains from HUG, 16 belong to patients with a suspected source of acquisition in the ICU, 1 from a patient with suspected cross transmission outside ICU and 2 from patients with nosocomial but unknown source of acquisition

Clinical isolates from the 16 patients with suspected ICU acquisition and 2 isolates from environmental sampling were sequenced. All isolates belonged to the sequence type 111 (ST-111). Additionally, 3 other clinical isolates (one from a patient with suspected cross transmission outside ICU and 2 from patients with unknown source of acquisition) were sequenced and belonged to the same cluster. Isolates were compared with 6 ST-111 strains from a cluster in a UK hospital [21] and 5 ST-111 strains from a French hospital [22]. The analysis of these 32 genomes showed 1112/5932 variable loci. Clustering was performed using a categorical coefficient and an UPGMA similarity tree was constructed (Fig. 3). Differences of 0–15 loci differences were observed among the 21 HUG isolates, confirming that the HUG cluster was different from the 11 other strains.

### Mitigation strategies

In September 2020, due to the detection of three new acquisition events and in regard to the conclusions of the environmental and genomic investigations with a clear evidence for a water reservoir, we implemented “water-free” patient care with removal of all sinks from patient rooms and of most of the sinks in medication preparation areas. Sinks were kept only in rooms dedicated to waste disposal and two sinks were kept in preparation rooms. All patient care-related activities that take place in the patient room and that would normally involve the use of tap water were adapted to a ‘water-free’ alternative (table in Additional file 1: Appendix). A bathing protocol using disposable chlorhexidine-free washing gloves and shampoo cap (SINAQUA®, WELCARE INDUSTRIES S.p.A.) was implemented. In September 2020, we also encouraged intensivists to re-assess on a case-by-case basis the indication for SOD and limit its use.

### Impact of the mitigation strategies and termination of the outbreak

After implementation of water-free patient care, no new acquisition event was detected in the ICU within the next 8 months (until May 2021). In January 2021, we discovered 4 new acquisition events of PA-VIM within HUG which occurred in a shared bedroom in an intermediate care unit hosting COVID-19 patients located in a different building. The first detected case had stayed in the ICU before but all screening samples collected during his stay in the ICU were negative. New environmental samples were collected in the sink located in the waste disposal room beside the ICU room the patient stayed and all were negatives.

## Discussion

We report here the successful control of a sink-related PA-VIM outbreak and the eradication of the water reservoir by introducing waterless patient care in an ICU of a tertiary care hospital in Switzerland.

The role of the hospital water environment as a reservoir and transmission pathway for hospital-acquired infections outbreaks has received increasing attention over the last decade [9, 10, 23]. Several outbreaks linked to the different water reservoirs (faucets, sinks, potable water, sink surfaces and wastewater drainage system) have been reported [24, 25], most frequently involving multidrug resistant Gram-negative bacteria including *Enterobacteriaceae* and non-fermentative Gram-negative such as *Pseudomonas sp, and Acinetobacter sp*. and other Gram-negative bacilli. These bacteria are well adapted to survive for a long time in the water environment and create biofilms, making them resistant to usual disinfection procedures. Water-related outbreaks occurred frequently in ICU settings, but have also been reported in burn units or neonatal intensive care units [9]. The population affected are frequently immunocompromised patients (e.g. haematological malignancies, stem cell transplants), as observed in the current outbreak. Furthermore, exposure to multiple medical devices has clearly been associated with water-related HAI [26, 27]. Several mechanisms and infection control breaches can lead to patient contamination from the water sources. Among them, poor sink designs, use of sinks for contaminated clinical disposal, storage of clean patient materials around sinks, blocked sewage pipe and waste pipes leaks have been reported [10, 19]. Hota et al. demonstrated, with the use of fluorescein injection into sinks, splash-back up to 1 m from the sink when the water was running [24]. In this scenario, biofilms spread from the sink trap to the sink drain and organisms are dispersed when impacted by water from the skin faucet. Gram-negative organisms dispersed can directly contaminate the patient or its environment in case of close contact between the patient bed and the sink (as it was the case in some patient rooms of our ICU), or can transiently contaminate the hands of healthcare workers during handwashing who can then contaminate the patient or its environment [29].

Several mitigation strategies have been reported with more or less success in outbreak termination and eradication of environmental water reservoirs [11]. Some engineering modifications can minimize contamination of surrounding surfaces and objects. For example, better sink design to reduce splashing, appropriate use of splash guards or barriers or rearrangement of the surrounding space with dedicated storage space more than 1 m from sinks have been proposed [30–32]. Nevertheless, major structural modifications are not always feasible. In our case, initial mitigations strategies implemented in the context of an endemic *S. marcescens* situation and which aimed to reduce colonisation of surrounding objects from sinks trough dedicated training of healthcare workers and modification of behaviors did not prevent the PA-VIM outbreak. Sinks in patient rooms were located less than 1 m from patient beds which left little room for architectural reorganization. Other interventions such as disinfection of sink drains attempted to reduce biofilms formation in sinks once colonized with multidrug-resistant organisms, but they have usually failed in reported outbreaks [11]. Novel technologies such as installation of devices that heat and vibrate on the exterior of traps or ozone might be promising; however, efficacy and cost-effectiveness still need to be demonstrated [33]. Implementation of waterless care activities was a successful measure in the context of our ICU, and has already been reported in a similar outbreak [24]. Hopman et al. reported that removal of the sinks from all patient rooms and the introduction of ‘water-free’ patient care in 2014 was associated with a statistically significant lower number of ICU patients that become colonized with Gram negative bacteria, including MDR Gram negative bacteria [34]. Nevertheless, this strategy is usually not implementable outside the ICU context and alternative solutions are urgently needed.

Selection pressure from administration of broad-spectrum antibiotics is well described as being an important contributor for acquisition [26]. Five patients in this outbreak received SOD before acquisition of the PSA-VIM. Emergence of colistin resistance among ESBL-producing *Klebsiella pneumoniae* isolates has been reported after introduction of Selective Digestive Tract Decontamination in an ICU [35]. Even if the potential benefits of SOD on protection against VAP are well described in the literature, we recommend in the context of a MDR outbreak, to re-assess the balance between benefits and risks and consider, at least temporarily, to suspend its administration.

We suspected one cross-transmission event in summer 2020 between the 1st and 2nd COVID-19 waves. As reported in an MRSA outbreak in an ICU during the SARS-CoV-1 epidemic [36] changes in infection control measures and personal protective equipment with universal gloving policy, among other factors, may have favoured this transmission event. Interestingly only one PA-VIM environmental acquisition was documented during the high activity of the 1st COVID-19 wave before water free patient care implementation.

WGS performed on 19 patient strains and 2 environmental strains showed that they all belonged to the ST-111 and had only 1–15 loci differences with wgMLST. This strongly suggest they are epidemiologically linked and belonged to the same chain of transmission [18].

Inferring potential transmission events solely based on epidemiological data proved challenging due to overlap of patients in the hospital. Considering that phylogenetically closely related isolates have a common ancestor and thus derive from a chain of transmission, the high power of discrimination of WGS allows to unravel outbreaks and decipher transmission events [37, 38].

In contrast to current conventional wisdom, our active patient screening policy did not help to completely stop the outbreak—it helped to detect cases and guide us towards an environmental reservoir. Thus, additional environmental screening and interventions are crucial and should be emphasized, in addition to conventional IPC measures.

A limitation of this study is the relatively short time lag of 8 months to evaluate the sustainability of the measures in stopping the outbreak, considering also that seasonality may have play a role.

## Conclusion

MDRO infections due to environmental sources is a major issue in ICU setting. We report an outbreak of PA-VIM in the ICU of a Swiss tertiary care hospital. Environmental and genomic investigations confirmed the aquatic source and the outbreak was controlled by implementation of waterless patient care, in addition to conventional infection control measures.

## Supplementary Information

**Additional file 1: Appendix**. Waterless alternatives implemented in the ICU for patient care-related actions.

## Data Availability

All supporting data will be made fully available.
