# Supplementary material for: Outbreak of Pseudomonas aeruginosa producing VIM carbapenemase in an intensive care unit and its termination by implementation of waterless patient care
Source: Crit Care. 2021 Aug 19;25:301. doi: 10.1186/s13054-021-03726-y (PMC8376114; doi:10.1186/s13054-021-03726-y)
Supplement: Supplementary file 1 — Additional file 1: Appendix. Waterless alternatives implemented in the ICU for patient care-related actions. [file 13054_2021_3726_MOESM1_ESM.docx]

Additional file 1: Appendix

Procedures implemented for applying waterless patient care

| Patient care-related actions | New ‘water-free’ method |
| --- | --- |
| Washing | Chlorhexidine-free washing gloves (SINAQUA ®, WELCARE INDUSTRIES S.p.A.) |
| Washing for patients with stools | Bottled water if necessary (Henniez SA, Switzerland) |
| Hair washing | Shampoo cap (SINAQUA ®, WELCARE INDUSTRIES S.p.A.) |
| Drinks | Bottled water (Henniez SA, Switzerland) |
| Canula care | Disposable materials |
| Dental care | Bottled water (Henniez SA, Switzerland) |
| Shaving | Electric shaving, or with warm bottled  water (Henniez SA, Switzerland) |
| Situation where hand washing with soap is recommanded | For situation where hand washing with soap is recommended (e.g *Clostridium difficile* infection) hand washing with soap is followed by hand rub with alcohol-based formulation |
